# Cathelicidin LL-37 Affects Surface and Intracellular Toll-Like Receptor Expression in Tissue Mast Cells

**DOI:** 10.1155/2018/7357162

**Published:** 2018-02-19

**Authors:** Justyna Agier, Ewa Brzezińska-Błaszczyk, Paulina Żelechowska, Magdalena Wiktorska, Jacek Pietrzak, Sylwia Różalska

**Affiliations:** ^1^Department of Experimental Immunology, Faculty of Health Sciences, Medical University of Lodz, Lodz, Poland; ^2^Department of Molecular Cell Mechanisms, Faculty of Health Sciences, Medical University of Lodz, Lodz, Poland; ^3^Department of Pharmaceutical Biochemistry and Molecular Diagnostics, Faculty of Pharmacy, Medical University of Lodz, Lodz, Poland; ^4^Department of Industrial Microbiology and Biotechnology, Faculty of Biology and Environmental Protection, University of Lodz, Lodz, Poland

## Abstract

Undoubtedly, mast cells take part in host defense against microorganisms as they are numerous at the portal of infection, they release many proinflammatory and antimicrobial mediators, and they express pattern recognition receptors, such as TLRs. These receptors play a key role in recognition and binding molecules associated with microorganisms and molecules associated with damage. Cathelicidins exhibit direct antimicrobial activities against a broad spectrum of microbes by perturbing their cell membranes. Accumulating evidence suggests a role for these molecules in supporting cell activation. We examined the impact of human cathelicidin LL-37 on tissue mast cell TLR expression and distribution. Depending on context, we show that LL-37 stimulation resulted in minor to major effects on TLR2, TLR3, TLR4, TLR5, TLR7, and TLR9 expression. Confocal microscopy analysis showed that, upon stimulation, TLRs may translocate from the cell interior to the surface and conversely. FPR2 and EGFR inhibitors reduced the increase in expression of selected receptors. We also established that LL-37 acts as a powerful inducer of CCL3 and ROS generation. These results showed that in response to LL-37, mast cells enhance the capability to detect invading pathogens by modulation of TLR expression in what may be involved FPR2 or EGFR molecules.

## 1. Introduction

Cathelicidins, the family of highly diverse antimicrobial peptides, are found in many mammalian species including rabbits, horses, pigs, rats, monkeys, cattle, and humans. These natural antibiotics are composed of 12–50 amino acid residues, and their molecular weight are in the range of 3 to 10 kDa. Peptides from the cathelicidin family have *α*-helix structure and are produced by neutrophils, endothelial cells, keratinocytes, macrophages, mast cells, NK cells, dendritic cells, and lymphocytes [1, 2]. In human, only one cathelicidin leucine-leucine-37 (LL-37) formed from the precursor protein hCAP18, is expressed. LL-37 and its precursor were found in different cells, tissues, and body fluids. Human cathelicidin is produced constitutively or synthesized in response to the presence of bacteria or their products. Expression of LL-37 may be adjusted by several endogenous factors, including inflammatory cytokines, growth factors, and active form of the vitamin D [3]. LL-37 exhibits direct antimicrobial activities against a broad spectrum of microbes, and it is an important effector molecule in the innate immunity mechanisms. Direct interaction of LL-37 is mainly involved in the disintegration of the microbial cell wall, cell membrane, and/or lipid envelope and consequently leads to cell death [4]. Moreover, human cathelicidin participates in the neutralization of endotoxin and reengineers bacterial biofilms [5, 6]. Apart from direct interaction with microbes, human cathelicidin can also exert immunomodulatory effects on cells involved in inflammatory processes [7]. LL-37 can act as a chemoattractant for neutrophils, monocytes, macrophages, eosinophils, and mast cells [8–10]. Human cathelicidin also stimulates the production of various chemokines and increases the expression of some chemokine receptors [11, 12]. Furthermore, LL-37 inhibits neutrophil programmed cell death by enhancing expression of the antiapoptotic BcL-xl protein and by blocking the activity of caspase-3 activity [13, 14]. Inhibition of neutrophil apoptosis may extend neutrophil lifespan and increased phagocytosis. What is more, LL-37 facilitates the formation of neutrophil extracellular traps [15]. Additionally, LL-37 upregulates the autophagy-related gene expression in macrophages and induces autophagosome formation to promote the killing of intracellular bacteria [16]. Although certain functions of LL-37 were revealed, there are no accepted specific cell receptors involved in LL-37 recognition and binding. Some information appears to indicate that the putative receptors for cathelicidins may be molecules from G protein-coupled receptor family, for example, *N*-formyl peptide receptor (FPR2) or Mas-related gene X2 (MrgX2). Other potential LL-37 receptors are purinergic receptor P2X_7_, epidermal growth factor receptor (EGFR), or C-X-C chemokine receptor type 2 (CXCR2) [3].

Mast cells play an important role in host defense against a variety of pathogens. An important prerequisite for the role of mast cells in defense mechanisms is their location. Mast cells are especially numerous in the skin and they are present under the epithelium of the respiratory tract, gastrointestinal tract, and urogenital tract. In the tissues, these cells are very numerous near the blood and lymph vessels. Such a strategic location, practically in the gate of infection, allows them to very easily and quickly have contact with the pathogens penetrating the body [17, 18]. Furthermore, it was documented that mast cells can produce antimicrobial peptides [19]. These cells can phagocytize and subsequently kill bacteria, via oxidative and nonoxidative systems, as well [20, 21]. What is important, mast cells support the development of antibacterial immunity by processing bacterial antigens through I and II MHC presentation [22, 23]. Finally, mast cell-derived cytokines and chemokines induce the development of inflammation at the site of pathogen entry [24].

Mast cells express receptors which play a crucial role in recognition and binding both pathogen-associated molecular patterns (PAMPs) and damage-associated molecular patterns (DAMPs) [25–27]. These specialized pattern recognition receptors (PRRs) are the transmembrane proteins once perforating the cell membrane (Toll-like receptors (TLRs) and C-type lectin receptors (CLRs)) or the membrane of the endosomes (TLRs). This group also includes the receptors present in the cytoplasm (RIG-I-like receptors (RLRs) and NOD-like receptors (NLRs)). Without any doubt, out of all PRRs, members of TLR family play a particularly significant role in initiating host defense against pathogens as these receptors recognize both wide ranges of PAMPs and various endogenous DAMPs released in response to infection.

Considering the important role of mast cells in the antimicrobial protection, it is essential to comprehend the regulation of TLR expression by these cells. Accordingly, we decided to evaluate the influence of cathelicidin LL-37, a key humoral factor of antimicrobial defense, on both surface and intracellular TLR expression in fully mature tissue mast cells.

## 2. Methods

### 2.1. Animals

Female albino Wistar rats weighing 200–250 g, aged 3-4 months, were used. All animal experimental procedures were carried out in strict accordance with the recommendations in the act on the protection of animals used for scientific or educational purposes. The experimental procedures were approved by the Local Ethics Committee for Experiments on Animals of the Medical University of Lodz (approval number 20/ŁB 740/2015).

### 2.2. Mast Cell Isolation

Mast cells were collected from peritoneal cavities of rats by density gradient separation technique, as previously described [28, 29]. After isolation, mast cells were counted and resuspended in an appropriate volume of cDMEM to obtain a concentration of 1.5 × 10^6^ cells/mL. Mast cells were prepared with purity > 98%, as determined by metachromatic staining with toluidine blue (Sigma-Aldrich). The viability of mast cells was over 98%, as estimated by trypan blue (Sigma-Aldrich) exclusion assay.

### 2.3. Quantitative RT-PCR

qRT-PCR was used to determine TLR2, TLR3, TLR4, TLR5, TLR7, and TLR9 mRNA levels. Purified mast cells suspended in cDMEM were stimulated with LL-37 (AnaSpec, Fremont, CA, USA) at a final concentration of 1 *μ*g/mL in a humidified atmosphere with 5% CO_2_ at 37°C for 1, 3, or 6 h. For control, mast cells were incubated under the same conditions without LL-37. qRT-PCR was conducted as previously described [10, 28]. The mRNA expression was corrected by normalization based on the transcript level of the housekeeping gene rat Actb.

### 2.4. Flow Cytometric Analysis (FACS)

For determination of constitutive and LL-37-induced cell surface of TLR2, TLR4, and TLR5 and intracellular localization of TLR3, TLR7, and TLR9, flow cytometry technique was used. Constitutive expression of TLRs was assessed in freshly isolated native mast cells (nonstimulated cells). To determine the intracellular localization of TLRs, mast cells were previously permeabilized with 0.1% saponin (Sigma-Aldrich) for 30 min at room temperature. LL-37-induced receptor expression was estimated on mast cells incubated with LL-37, at a final concentration of 1 *μ*g/mL, for 1, 3, or 6 h in a humidified atmosphere with 5% CO_2_ at 37°C. After that, mast cells were fixed with CellFIX solution (BD Bioscience, San Jose, CA, USA) for 15 min and washed twice with 1x PBS. Next, mast cells were resuspended in 1x PBS and stained for 1 h with appropriate antibodies (Santa Cruz Biotechnology Inc.). For control, mast cells were stained with isotype antibodies (R&D Systems Inc., Minneapolis, MN, USA). Primary antibody was not added to the sample to certify nonspecific binding of the secondary antibody. Specificities of antibodies were confirmed using blocking peptides. Cells were then washed with 1x PBS and incubated for 1 h with Alexa 488-conjugated secondary antibodies (Jackson ImmunoResearch Laboratories Inc., West Grove, PA, USA) in 1x PBS. After that, mast cells were washed twice with 1x PBS and resuspended in 1x PBS. Stained mast cell fluorescence was measured with FACS Canto II flow cytometer (BD Biosciences).

### 2.5. Confocal Microscopy

For determination of constitutive and LL-37-induced surface and intracellular distribution of TLR2, TLR3, TLR4, TLR5, TLR7, and TLR9, confocal microscopy was performed. To determine the subcellular localization of TLRs, mast cells were previously permeabilized with 0.1% saponin for 30 min at room temperature. Constitutive expression of TLRs was assessed in nonstimulated cells. LL-37-induced receptor expression was estimated in mast cells incubated with LL-37, at a final concentration of 1 *μ*g/mL, for 1, 3, or 6 h in a humidified atmosphere with 5% CO_2_ at 37°C. After that, mast cells were fixed with CellFIX solution for 15 min and washed twice with 1x PBS. Nonpermeabilized and permeabilized mast cells were stained for 1 h with appropriate antibodies (antibody dilution 1 : 100). Next, cells were washed with 1x PBS and incubated for 1 h with Alexa 488-conjugated secondary antibodies in 1x PBS. Finally, cells were washed and resuspended in 1x PBS. To confirm the specificity of primary antibody, isotype control was used. Primary antibody was not added to the sample to certify nonspecific binding of the secondary antibody.

To determine the contribution of the LL-37 potential receptor, P2X_7_R antagonist KN-62 (Tocris Bioscience, Bristol, UK), FPR2 antagonist PBP10 (Tocris Bioscience), and EGFR inhibitor AG1478 (Cayman Chemical, Ann Arbor, MI, USA) were used. Purified mast cells were pretreated with inhibitors or medium alone for 15 min at 37°C in a water bath with constant stirring, before the main procedure execution. KN-62 and PBP10 were used at concentrations of 1 *μ*M, and AG1478 was used at a concentration of 5 *μ*M. After that, cells were washed and incubated with LL-37 at 1 *μ*g/mL for 3 h. The concentrations of all applied inhibitors were chosen in the preliminary experiments, in accordance with the manufacturer's instructions, and neither of the inhibitors affected mast cell viability, as examined by trypan blue exclusion assay. For control, mast cells were incubated with DMSO (Sigma-Aldrich) under the same conditions (vehicle-treated control). The same staining protocol was used as above.

To indicate ROS generation, mast cells were incubated with LL-37 at 1 *μ*g/mL or medium alone for 30 or 60 min in a humidified atmosphere with 5% CO_2_ at 37°C. Indicator for ROS, H_2_DCFDA (Thermo Fisher Scientific), was used at a concentration of 2 *μ*M for 10 min. After that, cells were washed and resuspended in 1x PBS.

Confocal microscopy method was conducted according to a previously described procedure [30]. All signals obtained from confocal microscopy were validated with profile view image analysis and the diagrams presenting intensity values placed beside each microphotograph. The mean fluorescence intensity (expressed in arbitrary units AU) were calculated for each of the samples. The calculations were done for at least 40 different points randomly selected in compartments with receptor expression.

### 2.6. ELISA

Purified mast cells suspended in the medium for rat mast cells (containing 137 mM NaCl, 2.7 mM KCl, 1 mM CaCl_2_, 1 mM MgCl_2_, 10 mM HEPES buffer, 5.6 mM glucose, and 1 mg/mL BSA) were incubated with LL-37 at final concentrations of 1, 10, 20, or 40 *μ*g/mL, mouse anti-rat IgE (AbD Serotec, Oxford, UK) at a final concentration of 5 *μ*g/mL (positive control) or buffer alone (nonstimulated cells) in a water bath for 120 min at 37°C with constant stirring. The supernatants were collected by centrifugation. The concentration of CCL3 in supernatants was evaluated by ELISA kit (Cloud-Clone Corp., Katy, TX, USA), according to the manufacturer's instructions. The sensitivity of the assay was < 59 pg/mL.

### 2.7. Statistical Analysis

The statistical analysis of experimental data was performed using Statistica 13 software (Statsoft Inc., USA). Data are presented as the mean ± SD. Normality of distribution was tested with the Shapiro-Wilk test. All comparisons between groups were carried out by using Student's *t*-test for small groups or one-way ANOVA. Differences were considered significant at *P* < 0.05 and are labeled with an asterisk (∗) on each graph.

## 3. Results

### 3.1. Effect of LL-37 on TLR mRNA Expression

**Figure 1:**
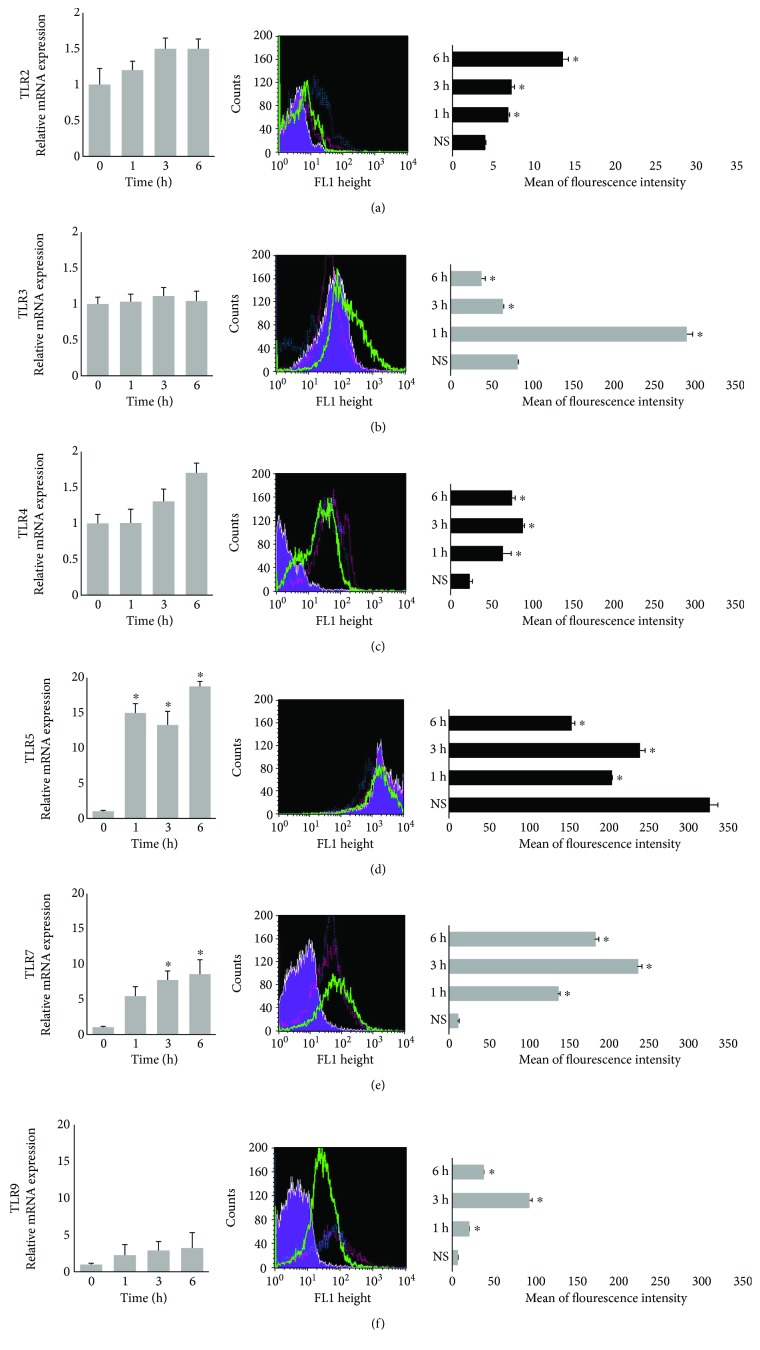
mRNA and protein levels of (a) TLR2, (b) TLR3, (c) TLR4, (d) TLR5, (e) TLR7, and (f) TLR9 in resting and LL-37-stimulated mast cells. Mast cells were incubated with LL-37 at a final concentration of 1 *μ*g/mL or medium alone (nonstimulated (NS)) for 1 h, 3 h, or 6 h. Left panel: TLR mRNAs expression was assessed using qRT-PCR. The expression of receptor mRNAs was corrected by normalization based on the transcript level of the housekeeping gene rat Actb. Results are the mean ± SD of three experiments performed in duplicate (*n* = 6). Middle panel: TLR protein expression assessed by flow cytometry. The results shown are representative of three independent experiments. Shaded tracings: TLRs expression in nonstimulated cells; open tracings: TLRs expression in cells after LL-37 stimulation for 1 h (green), 3 h (red), and 6 h (violet). Right panel: flow cytometry analysis of surface (s) TLR2, intracellular (i) TLR3, sTLR4, sTLR5, iTLR7, and iTLR9 expression. The data represent the mean of fluorescent intensity ± SD of three experiments performed in duplicate (*n* = 6). Comparisons between groups were carried out by using Student's *t*-test for small groups. Differences were considered significant at *P* < 0.05 and are labeled with an asterisk (∗) on each graph.

We first examined the expression of TLR mRNAs by mature rat mast cells in response to LL-37. TLR2, TLR3, TLR4, TLR5, TLR7, and TLR9 transcripts were analyzed after 1, 3, and 6 h of stimulation with 1 *μ*g/mL LL-37 by qRT-PCR. We found that mature mast cells constitutively express mRNA for all studied TLRs (Figure 1, left panel). LL-37 stimulation did not affect mRNA levels of TLR2, TLR3, TLR4, and TLR9. However, 1 h stimulation with LL-37 caused an increase in TLR5 mRNA expression (Figure 1(d)), and the expression of TLR7 mRNA was significantly higher at 3 and 6 h (Figure 1(e)). There were no statistically significant differences between means as determined by one-way ANOVA in the case of TLR2, TLR3, TLR4, and TLR9. We noticed statistically significant differences determined by one-way ANOVA in the case of TLR5 (*P* = 0.039) and TLR7 (*P* = 0.025).

### 3.2. Effect of LL-37 on TLR Protein Expression

We were next interested in determining whether LL-37 stimulation influences TL receptor protein expression. Mast cells were incubated with LL-37 at a final concentration of 1 *μ*g/mL or medium alone for 1, 3, or 6 h. As shown in the middle panel of Figure 1, both resting and LL-37-stimulated mast cells express all studied TLR proteins. Using flow cytometry, we evaluated the changes in TLR protein expression levels induced by LL-37 (Figure 1, right panel). We examined surface TLR2, TLR4, and TLR5 expression and intracellular TLR3, TLR7, and TLR9 levels. As shown in Figure 1(a), LL-37 caused a significant increase in TLR2 protein level in a time-dependent manner compared with nonstimulated cells. Mast cell stimulation with LL-37 resulted in an increase at 1 h of incubation in TLR3 intracellular expression (Figure 1(b)). Longer incubation resulted in decreased TLR3 expression compared to nonstimulated cells. Likewise, LL-37 induced enhancement of TLR4 protein level and the intensity of the signals was the highest after 3 h of stimulation (Figure 1(c)). LL-37 also affected TLR5 expression level (Figure 1(d)). Peptide stimulation resulted in a decrease of the signal in the cell membrane after 1 h which kept up in each time of incubation. We observed that intensity of intracellular fluorescence for TLR7 and TLR9 was considerably higher after incubation with LL-37 (Figures 1(e) and 1(f)). There were no statistically significant differences between means as determined by one-way ANOVA in the case of TLR2, TLR3, TLR4, and TLR7. We established statistically significant differences determined by one-way ANOVA in the case of TLR5 (*P* = 0.026) and TLR9 (*P* = 0.029).

**Figure 2:**
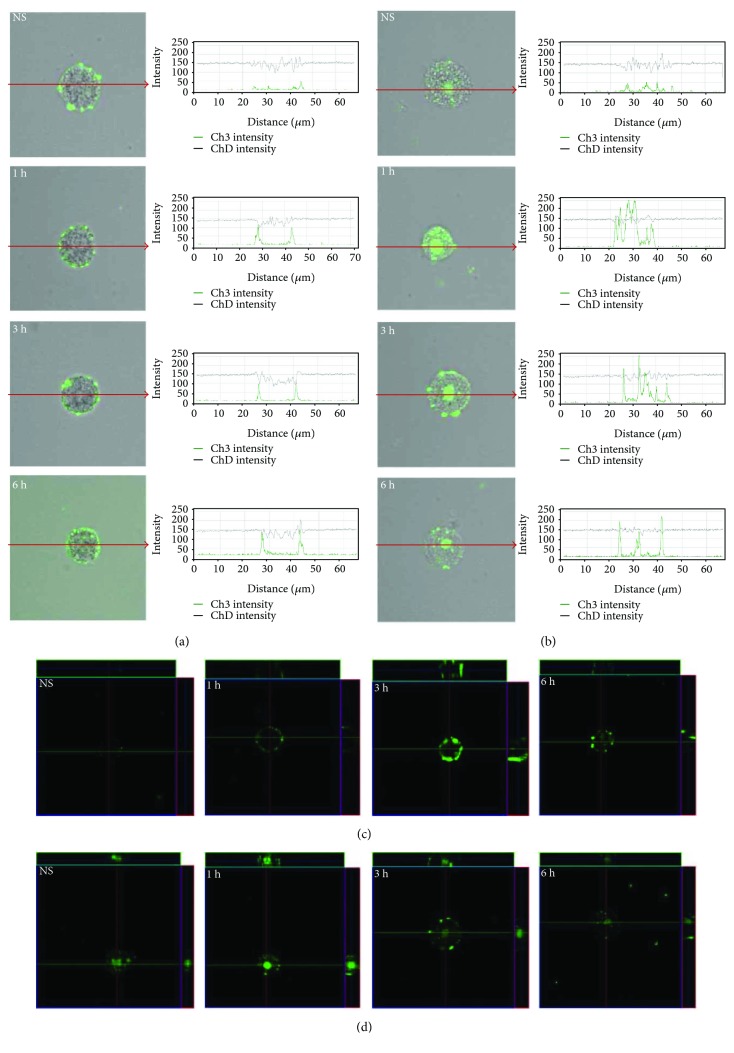
Effect of LL-37 stimulation on TLR2 expression in mast cells. Mast cells were incubated with LL-37 at a final concentration of 1 *μ*g/mL or medium alone (NS) for 1 h, 3 h, or 6 h. Representative images showing TLR2 cellular localization analyzed by confocal microscopy in (a, c) non- and (b, d) permeabilized cells. Single confocal sections (midsection of cells) reveal the surface and intracellular presence of TLR2. The signal was visualized with green Alexa 488. Fluorescence intensity diagrams showing the distribution of fluorescence in cells were mounted.

To assess location and distribution of TLRs, confocal microscopy technique was used. Mast cells were stained for surface and intracellular expression of all studied receptors. Isotype control and control for nonspecific binding of the secondary antibody confirmed the specificity of antibodies (data not shown). The changes in TLR2 expression are shown in Figure 2. The confocal microscopy and image analysis confirmed the presence of TLR2 on the surface and clearly showed intracellular expression of this receptor in unstimulated cells. Mast cell stimulation with LL-37 resulted in an increase of TLR2 surface expression level in a time-dependent manner. The strong TLR2 intracellular signal in the perinuclear region was found at 1 h and 3 h. After 6 h of incubation, the intensity of the signals from nucleus envelope was weaker but also detectable. The strong TLR2 intracellular signal in the perinuclear region was found at 1 h (230.4 ± 19.2 fluorescence intensity arbitrary units (AU)) and 3 h (217.0 ± 25.4 AU) in comparison to unstimulated cells (48.4 ± 5.7 AU); *P* < 0.001. After 6 h of incubation, the intensity of the signals from nucleus envelope was weaker but also detectable (161.8 ± 20.9 AU); *P* < 0.001.

**Figure 3:**
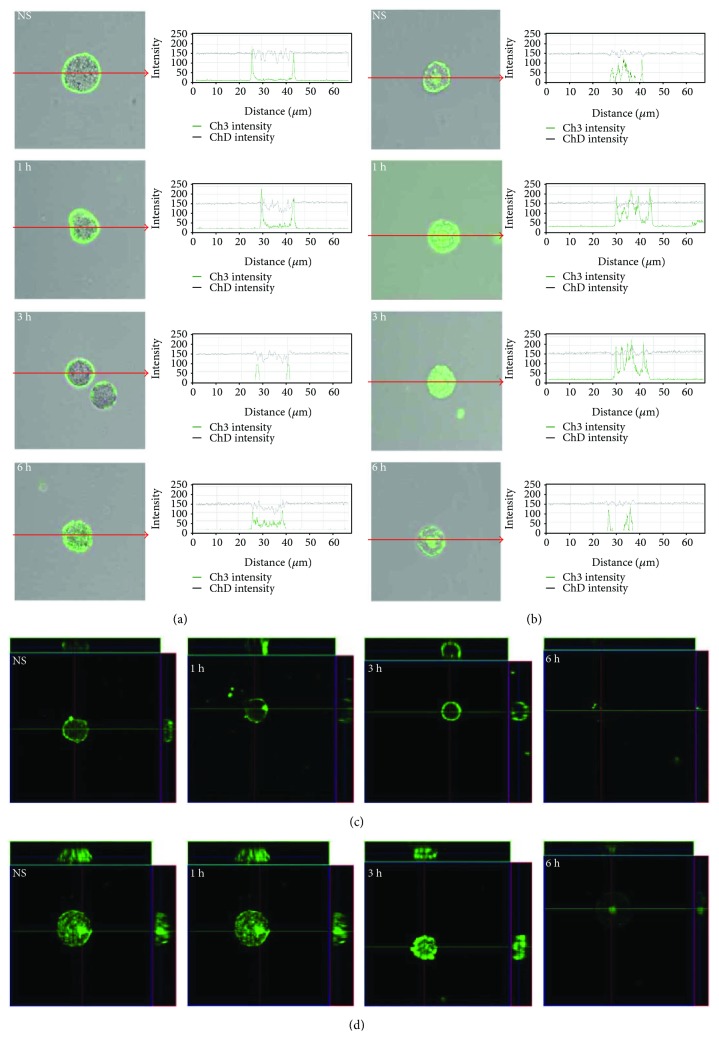
Effect of LL-37 stimulation on TLR3 expression in mast cells. Mast cells were incubated with LL-37 at a final concentration of 1 *μ*g/mL or medium alone (NS) for 1 h, 3 h, or 6 h. Representative images showing TLR3 cellular localization analyzed by confocal microscopy in (a, c) non- and (b, d) permeabilized cells. Single confocal sections (midsection of cells) reveal the surface and intracellular presence of TLR3. The signal was visualized with green Alexa 488. Fluorescence intensity diagrams showing the distribution of fluorescence in cells were mounted.

**Figure 4:**
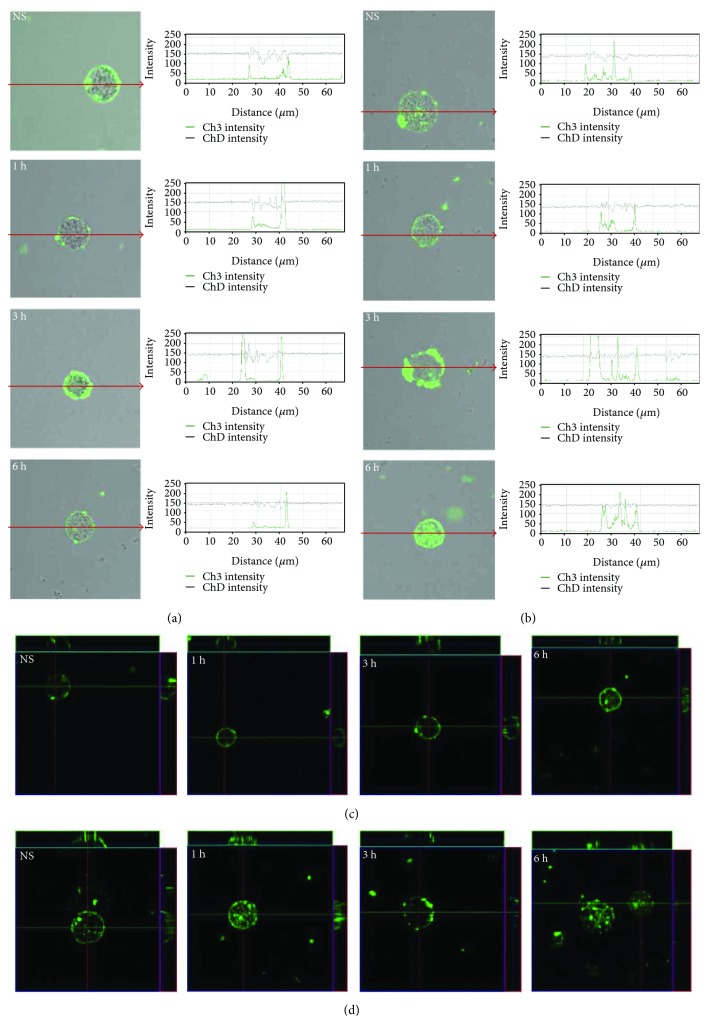
Effect of LL-37 stimulation on TLR4 expression in mast cells. Mast cells were incubated with LL-37 at a final concentration of 1 *μ*g/mL or medium alone (NS) for 1 h, 3 h, or 6 h. Representative images showing TLR4 cellular localization analyzed by confocal microscopy in (a, c) non- and (b, d) permeabilized cells. Single confocal sections (midsection of cells) reveal the surface and intracellular presence of TLR4. The signal was visualized with green Alexa 488. Fluorescence intensity diagrams showing the distribution of fluorescence in cells were mounted.

**Figure 5:**
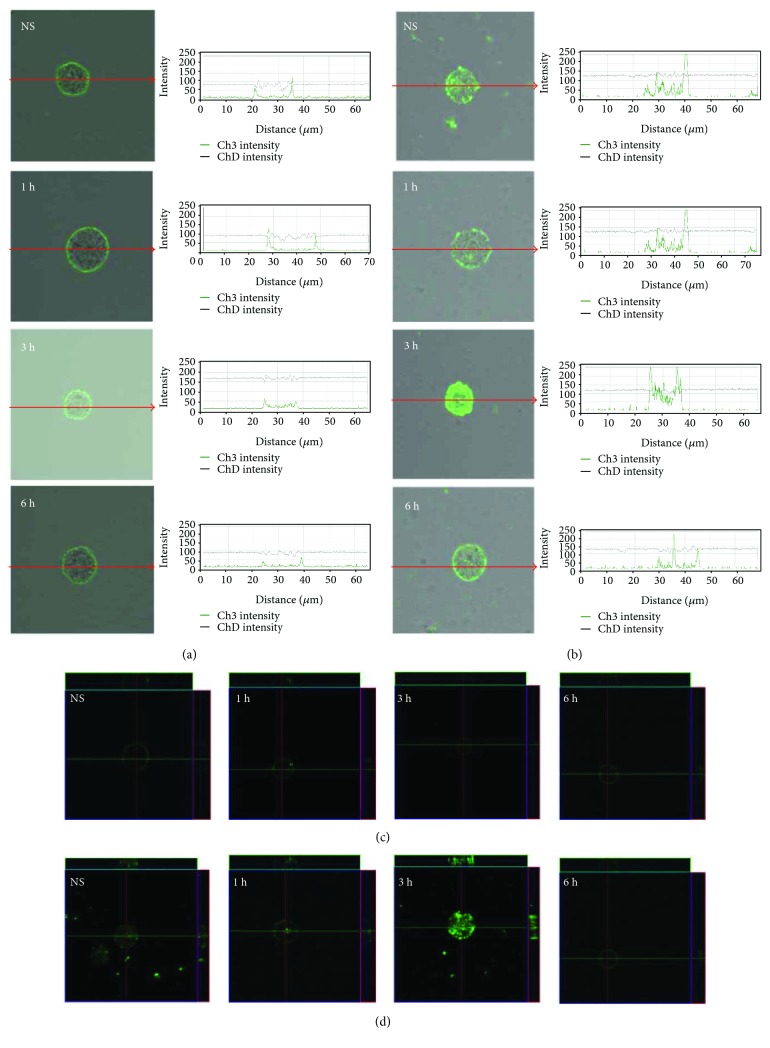
Effect of LL-37 stimulation on TLR5 expression in mast cells. Mast cells were incubated with LL-37 at a final concentration of 1 *μ*g/mL or medium alone (NS) for 1 h, 3 h, or 6 h. Representative images showing TLR5 cellular localization analyzed by confocal microscopy in (a, c) non- and (b, d) permeabilized cells. Single confocal sections (midsection of cells) reveal the surface and intracellular presence of TLR5. The signal was visualized with green Alexa 488. Fluorescence intensity diagrams showing the distribution of fluorescence in cells were mounted.

The expression of TLR3 by mast cells is shown in Figure 3. By using immunocytochemical staining, we visualized that fluorescence is predominantly associated with the nuclear envelope and endoplasmic reticulum. Confocal microscopy and image analysis revealed that in resting mast cells, TLR3 is also located on the cell surface. Mast cell stimulation with cathelicidin resulted in an increase of signals both in the cell membrane (188.9 ± 31.0 AU) and intracellular regions (169.9 ± 8.0 AU) after 1 h which was also documented by intensity diagrams beside each microphotograph; *P* < 0.001. In turn, incubation with LL-37 for an extended time caused a decrease in TLR3 intracellular expression (125.2 ± 11.6 AU) in about 18% compared with nonstimulated cells (151.1 ± 10.9 AU); *P* < 0.001. Mast cell stimulation with cathelicidin resulted in an increase of signals both in the cell membrane and intracellular regions after 1 h which was documented by intensity diagrams beside each microphotograph. In turn, incubation with LL-37 for an extended time caused a decrease in TLR3 intracellular expression in about 50% compared with nonstimulated cells. Above observations are in good agreement with flow cytometric analysis. As demonstrated in Figure 4, TLR4 is mainly located on the cell surface. In the cell interior of nonstimulated permeabilized cells, we observed only inconsiderable fluorescence signal associated with the nuclear envelope. Mast cell treatment with LL-37 caused an enhancement of TLR4 on the cell surface, which was confirmed by intensity diagram analysis. The intensity of the signals was the highest after 3 h of stimulation (227.7 ± 36.6 AU) in comparison to nonstimulated cells (80.4 ± 14.1 AU); *P* < 0.001. The intensity of the signals was the highest after 3 h of stimulation. The expression of TLR5 by mast cells is shown in Figure 5. We have exhibited that the significant receptor pool is stored not on the surface but intracellularly. LL-37 strongly increased expression of TLR5 after 3 h exposure inside the cell. LL-37 strongly increased expression of TLR5 after 3 h exposure inside the cell (168.3 ± 46.5 AU) in comparison to nonstimulated cells (75.1 ± 26.5 AU); *P* < 0.001.

**Figure 6:**
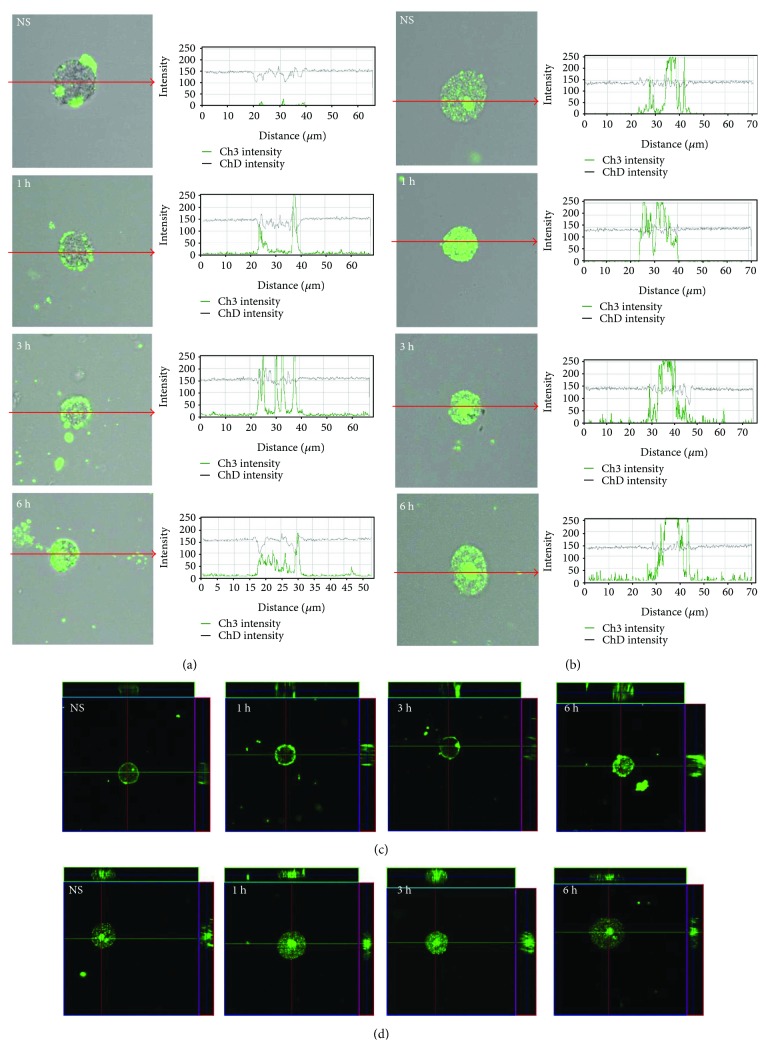
Effect of LL-37 stimulation on TLR7 expression in mast cells. Mast cells were incubated with LL-37 at a final concentration of 1 *μ*g/mL or medium alone (NS) for 1 h, 3 h, or 6 h. Representative images showing TLR7 cellular localization analyzed by confocal microscopy in (a, c) non- and (b, d) permeabilized cells. Single confocal sections (midsection of cells) reveal the surface and intracellular presence of TLR7. The signal was visualized with green Alexa 488. Fluorescence intensity diagrams showing the distribution of fluorescence in cells were mounted.

**Figure 7:**
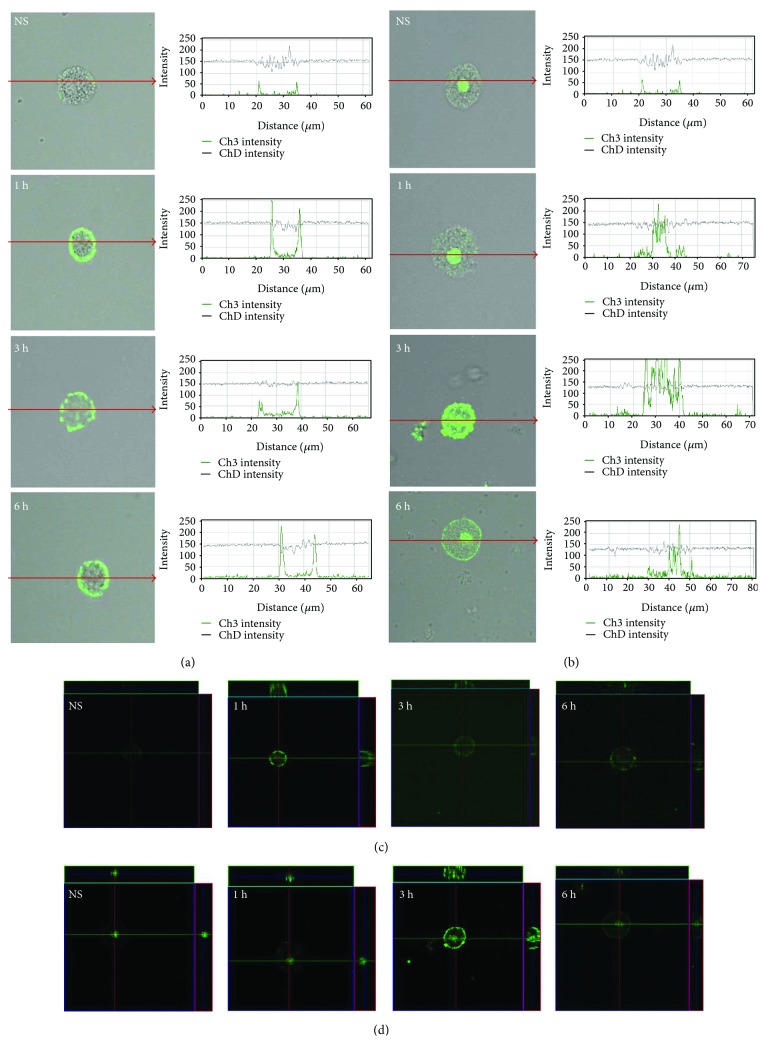
Effect of LL-37 stimulation on TLR9 expression in mast cells. Mast cells were incubated with LL-37 at a final concentration of 1 *μ*g/mL or medium alone (NS) for 1 h, 3 h, or 6 h. Representative images showing TLR9 cellular localization analyzed by confocal microscopy in (a, c) non- and (b, d) permeabilized cells. Single confocal sections (midsection of cells) reveal the surface and intracellular presence of TLR9. The signal was visualized with green Alexa 488. Fluorescence intensity diagrams showing the distribution of fluorescence in cells were mounted.

Single confocal sections, supplemented with fluorescence intensity diagrams, showed that in freshly isolated mast cells, TLR7 is localized mainly in the cell interior (Figure 6). LL-37 induced TLR7 expression increase in the cell interior which was evidenced by fluorescence intensity diagrams. In the presence of antimicrobial peptide, the intensity of the signals from endoplasmic reticulum was comparable at 1, 3, and 6 h. In the presence of antimicrobial peptide, the intensity of the signals from endoplasmic reticulum was comparable at 1 h (237.8 ± 34.5 AU), 3 h (247.4 ± 13.6 AU), and 6 h (250.2 ± 8.7 AU); *P* < 0.001. The changes in TLR9 expression level are shown in Figure 7. In resting mast cells, TLR9 is predominantly in the nucleus envelope and a weak signal was obtained from the cell surface. The receptor was significantly upregulated upon incubation with LL-37. Image analysis revealed that intensity of cell surface fluorescence was considerably higher at 1 h and inside the cell at 3 h. The receptor was significantly upregulated upon incubation with LL-37. Image analysis revealed that intensity of cell surface fluorescence was considerably higher at 1 h (218.7 ± 28.7 AU) in comparison to nonstimulated cells (35.3 ± 6.3 AU); *P* < 0.001. The same tendency was noticed for receptors localized in nucleus envelope at 3 h and the mean intensity amounted (231.1 ± 33.5 AU) in comparison to nonstimulated cells (97.9 ± 27.4 AU); *P* < 0.001.

### 3.3. Effect of LL-37 on Mast Cell Activity

**Figure 8:**
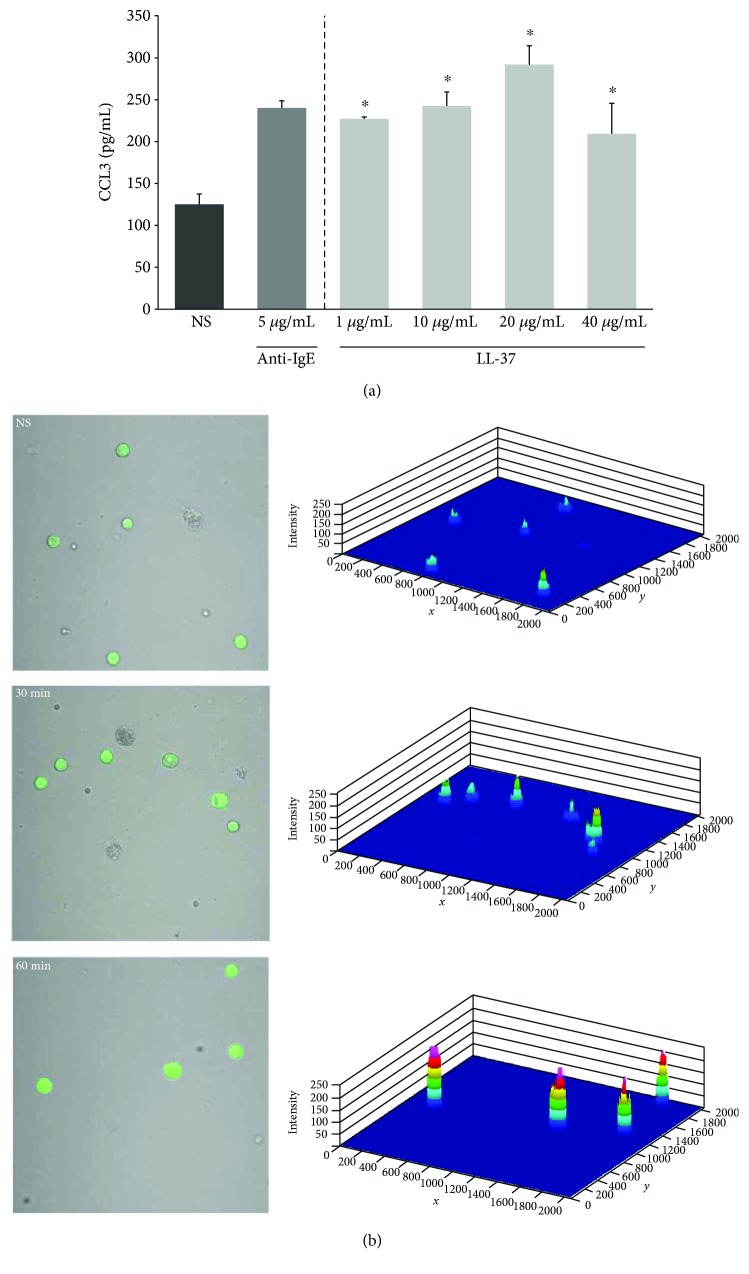
Effect of LL-37 on (a) CCL3 and (b) ROS production. For CCL3 measurement, mast cells were incubated with different concentrations of LL-37, anti-IgE at 5 *μ*g/mL (positive control), or medium alone (NS) for 2 h. Results are the mean ± SD of four independent experiments (*n* = 8). Comparisons between groups were carried out by using Student's *t*-test for small groups. Differences were considered significant at *P* < 0.05 and are labeled with an asterisk (∗) on each graph. To evaluate ROS generation, mast cells were incubated with 1 *μ*g/mL LL-37 for 30 or 60 min or medium alone (NS). Indicator for ROS, H_2_DCFDA, was used at a concentration of 2 *μ*M for 10 min.

Next, we sought to determine whether LL-37 stimulation induces the mast cell proinflammatory response. To this end, mast cells were stimulated with LL-37 at the concentrations of 1, 10, 20, and 40 *μ*g/mL for 2 h, using medium alone or anti-IgE as negative and positive controls, respectively. After incubation, the levels of CCL3 in supernatants were determined by the specific ELISA kit. The results of these experiments are shown in Figure 8(a). We stated that mast cell stimulation with LL-37 induced significant synthesis of CCL3, comparable to anti-IgE-induced chemokine generation. Chemokine secretion in response to mast cell stimulation with LL-37 used at 20 *μ*g/mL was the highest and up to 292.3 ± 22.48 pg/mL. We established statistically significant differences determined by one-way ANOVA (*P* = 0.025). LL-37-induced ROS generation by mast cells was evaluated by confocal microscopy. Mast cells were incubated with cathelicidin at the concentration of 1 *μ*g/mL for 30 or 60 min. As shown in Figure 8(b), LL-37 increased the basal level of ROS both after 30 and 60 min of stimulation in about 2- and 4-fold, respectively.

### 3.4. Involvement of Surface Molecules in LL-37-Induced Mast Cell Response

**Figure 9:**
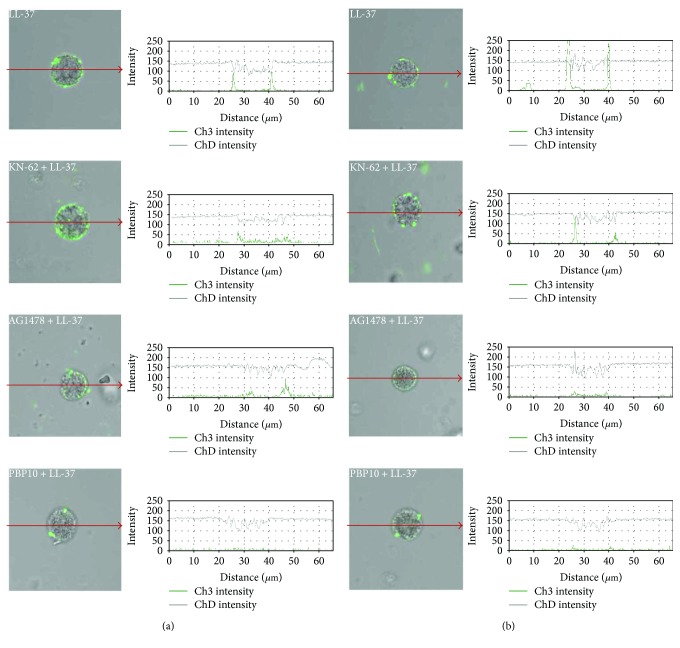
Effect of P2X_7_, FPR2, and EGFR inhibitors on LL-37-induced (a) TLR2 and (b) TLR4 expression. Mast cells were preincubated with P2X_7_ antagonist KN-62 at 1 *μ*M, FPR2 antagonist PBP10 at 1 *μ*M, or EGFR inhibitor AG1478 at 5 *μ*M or medium alone for 15 min prior to stimulation with LL-37 at 1 *μ*g/mL for 3 h. Representative images showing TLR cellular localization analyzed by confocal microscopy in nonpermeabilized cells. Single confocal sections (midsection of cells) reveal the presence of receptors. The signal was visualized with green Alexa 488. Fluorescence intensity diagrams showing the distribution of fluorescence in cells were mounted.

Our goal here was to elucidate the involvement of surface molecules P2X_7_R, FPR2, and EGFR in the activation of mast cells by LL-37. Therefore, we performed inhibitory experiments in which P2X_7_R antagonist KN-62, FPR2 antagonist PBP10, and EGFR inhibitor AG1478 were used. The signals obtained from confocal microscopy were validated with profile view image analyses (besides each microphotograph). We documented that mast cell pretreatment with the FPR2 antagonist and EGFR inhibitor noticeably and significantly suppressed LL-37-mediated TLR2 and TLR4 upregulation (Figure 9). Mast cell preincubation with KN-62 did not affect cathelicidin-induced TLR2 and TLR4 expression changes. Vehicle control showed that inhibitor dissolvent had no impact on the obtained results.

## 4. Discussion

The course of the immune response to infection depends, among others, on many humoral factors, that is, complement system proteins, acute phase proteins, and alarmins including heat shock proteins, cytokines, interferons, and chemokines. The above-mentioned agents synthesized by many cells, in direct and indirect ways, modulate defense processes directed against pathogens. A significant group of humoral factors involved in defense mechanisms is the antimicrobial peptides, including primarily cathelicidins and defensins. Despite the multidirectional activities of cathelicidins, accumulating evidence suggests a role for these molecules in supporting cell activation.

It is well established that mast cells take part in defense mechanisms. Therefore, it is important to understand the influence of humoral factors involved in regulation of immune response to pathogen invasion on mast cell phenotype and activity. That is why we decided to assess the impact of cathelicidin LL-37 on the expression of TLRs, the molecules that play a pivotal role in innate immunity, in mast cells. Until today, TLR expression, at transcript and protein levels, was documented in different mast cell lines, such as HMC-1, KU812, LAD, and MC/9, as well in various mast cell differentiated *in vitro*, including BMMCs, CBMCs, FSMCs, HCMCs, and PBMCs [25, 31]. In this study, we evaluated the expression of TLRs in matured *in vivo* rat mast cells isolated from the peritoneal cavity, that is, connective tissue-type mast cells. We established that these cells constitutively express mRNAs and proteins of all studied receptors. We clearly stated that TLR4 is located on the cell surface and that TLR7 and TLR9 are present mainly in the cell interior. It is worth pointing out that in resting mast cells, TLR2 and TLR5 were detected not only in the cell surface but also in the perinuclear region and that, although the TLR3 protein was predominantly found in the intracellular compartment, it is expressed in the cell surface, as well. Considering the important role of mast cells in host antimicrobial defense, these observations, particularly regarding TLR2, TLR3, and TLR5, are intriguing as compartmentalization of different TLRs imposes not only which ligands are recognized but also the type of cell response.

Furthermore, we established that TLR expression and distribution in mast cells are strongly modulated by cathelicidin LL-37. Confocal imaging indicated that this peptide significantly upregulates TLR2 and TLR4 expression at the cell surface probably by inducing translocation of intracellular molecules to the cell membrane. Treatment with LL-37 caused the increase of TLR9 expression both at the cell surface and cell interior, as well. Microscopy analysis showed that LL-37 can cause translocation of TLR3 from the plasma membrane to the cytoplasm, as this peptide induced a transient increase of TLR3 in cell interior while surface expression of this receptor was decreased. Moreover, we documented that LL-37 treatment enhanced the intracellular expression of TLR5 and TLR7, presumably by increased synthesis of new molecules.

Considering the significance of TLRs in the development of innate immunity processes, it seems to be of great importance to comprehend factors that influence TLR expression; however, there is little information on this subject. It was shown that treatment of murine macrophage cell line RAW264.7 with IL-2, IL-15, IL-1*β*, IFN-*γ*, and TNF increases TLR2 mRNA expression [32]. Kurt-Jones et al. [33] stated that GM-SCF stimulation also upregulates TLR2 mRNA level in neutrophils. In turn, IFN-*γ* and M-CSF upregulate surface expression both TLR2 and TLR4 proteins on human peripheral blood monocytes [34]. It was also indicated that some cytokines, including proinflammatory cytokines, can modulate the expression of TLRs in mast cells; however, the data are scarce. Okumura et al. [35] stated that IFN-*γ* upregulates mRNA and both surface and intracellular protein levels of TLR4 in human mast cells. Yang et al. [36] established that IL-12 induces a significant increase in mRNA and protein expression of TLR2 and TLR4 in P815 mast cell line. Previously, we noticed that IL-6 treatment of rat peritoneal mast cells causes an increase in TLR4 protein expression, and exposure to chemokine CCL5 results in decreased expression of both TLR2 and TLR4 proteins [37]. It was also documented that GM-SCF provokes upregulation of TLR3 and TLR7 mRNAs and proteins expression in P815 cells [38]. Only Yoshioka et al. [39] evaluated the effect of LL-37 on TLR4 expression in mast cells and noticed that this cathelicidin upregulates TLR4 expression in LAD2 cells.

There are data that LL-37 can directly activate mast cells to proinflammatory activity. It was documented that this peptide causes degranulation of both immature [40, 41] and mature [42–44] mast cells, as examined by histamine and *β*-hexosaminidase release assessment. LL-37 activates LAD2 cells to LTC_4_ and PGE_2_ production and release [44] and stimulates mast cells to the production of certain cytokines, such as IL-1*β*, IL-2, IL-4, IL-5, IL-6, IL-31, TNF, GM-CSF, and chemokine CCL4 [40, 41, 44]. Previously, we demonstrated that cathelicidin LL-37 stimulates rat tissue mast cells to histamine secretion and TNF and IL-6 release and induces mRNA expression of some proinflammatory cytokines and chemokines, including IL-1*β*, CCL2, and CCL3, as well [10]. Likewise, in this paper, we documented that LL-37 activates mast cells to chemokine CCL3 synthesis and ROS generation.

To date, it has not been entirely clear what are the exact cathelicidin concentrations in physiological and pathological conditions. In healthy individuals, the LL-37 levels were as low as 39.9 pg/mL in sputum [45], 1–4 ng/mL in tracheal aspirate samples obtained from new-born children [46], 30.5 ng/mL in saliva [47], and 1.1 and 1.7 ng/mL in plasma of neonatal and maternal blood, respectively [48, 49]. Admittedly, in healthy adult individuals, the serum concentration of this peptide is within the range 1-2 ng/mL [50, 51]; however, Jeng et al. [52] noticed that LL-37 plasma level was about 27.2 ng/mL. Furthermore, concentrations of LL-37 in sweat and BAL of healthy infants are significantly higher and amount up to 4.47 *μ*g/mL [53] and 4.8 *μ*g/mL, respectively [54]. In our studies conducted *in vitro*, we used LL-37 at 1 *μ*g/mL to assess antimicrobial peptide impact on TLR expression and concentrations from 1 to 40 *μ*g/mL to determine whether LL-37 stimulation induces the mast cell CCL3 generation. Therefore, we can assume that concentrations of LL-37 applied in our studies reflect the cathelicidin physiological levels.

Up to date, it is not clear which surface molecule acts as a functional receptor for cathelicidin LL-37. There are data that LL-37 may act through FPR2, as this molecule is involved in the LL-37-mediated migration of neutrophils, monocytes, CD4^+^ T lymphocytes, and eosinophils [8, 9]; LL-37-induced suppression of neutrophil apoptosis [14]; and promotion of monocyte adhesion [55]. Sun et al. [56] documented that this peptide stimulates eosinophils to cysLT production acting via FPR2 molecules. Some information seems to suggest that LL-37 can induce cell response by interacting with tyrosine kinase receptors such as EGFR. Tjabringa et al. [57] documented that LL-37 peptide, via EGFR, activates epithelial cells to CXCL8 synthesis, and Shaykhiev et al. [58] noticed that LL-37 stimulates proliferation and migration of airway epithelial cells acting through EGFR molecule. It was also stated that LL-37, via EGFR, induces keratinocyte migration [59]. Few data appear to indicate that also P2X_7_ purinergic receptor can be involved in LL-37-induced cell activation. This peptide, via P2X_7_ molecule, stimulates monocytes for IL-1*β* synthesis [60] and activates fibroblasts to CXCL8 production [61] and macrophages to LTB_4_ and TXA_2_ synthesis [62]. Zhang et al. [63] provided evidence that LL-37 may act as a functional ligand for CXCR2 on human neutrophils. Likewise, there is no information on which mast cell surface molecule acts as a receptor for cathelicidin LL-37. It has been reported that GPCRs mediate LL-37-induced signal transduction pathway in mast cells [43, 64]. Subramanian et al. [40] and Yu et al. [65] indicated that this peptide stimulates mast cells by MrgX2 receptor. It was also suggested that LL-37 can act via FPR2 or P2X_7_, as Yoshioka et al. [39] indicate expression of these molecules on LAD2 cells. In this study, we documented that mast cell pretreatment with the FPR2 antagonist and EGFR inhibitor noticeably and significantly suppressed LL-37-mediated TLR2 and TLR4 upregulation. Thus, we can hypothesize that FPR2 or EGFR molecules, but not P2X_7_, might be the functional receptors for LL-37 on rat tissue mast cells.

## 5. Conclusion

In conclusion, we have provided evidence that LL-37 directly influences TLR level and distribution in mature tissue-resident mast cells. This peptide causes enhancement of TLR2, TLR4, and TLR9 on the mast cell surface and TLR3, TLR5, and TLR7 in the cell interior. TLRs play a crucial role in the early innate immune response as they recognize highly conserved microbial structural motifs as well as different endogenous molecules synthesized in response to microbial infection. Hence, LL-37 might enhance mast cell capability to detect invading pathogens and host DAMPs by modulation of TLR expression. These findings seem to be vital in view of mast cells' importance in host defense processes developed in response to infection.
